# Electrical characterisation of deep level defects in Be-doped AlGaAs grown on (100) and (311)A GaAs substrates by MBE

**DOI:** 10.1186/1556-276X-6-180

**Published:** 2011-02-28

**Authors:** Riaz H Mari, Muhammad Shafi, Mohsin Aziz, Almontaser Khatab, David Taylor, Mohamed Henini

**Affiliations:** 1 School of Physics and Astronomy, University of Nottingham, Nottingham NG7 2RD, UK; 2 Nottingham Nanotechnology and Nanoscience Center, University of Nottingham, Nottingham NG7 2RD, UK

## Abstract

The growth of high mobility two-dimensional hole gases (2DHGs) using GaAs-GaAlAs heterostructures has been the subject of many investigations. However, despite many efforts hole mobilities in Be-doped structures grown on (100) GaAs substrate remained considerably lower than those obtained by growing on (311)A oriented surface using silicon as *p*-type dopant. In this study we will report on the properties of hole traps in a set of *p*-type Be-doped Al_0.29_Ga0_.71_As samples grown by molecular beam epitaxy on (100) and (311)A GaAs substrates using deep level transient spectroscopy (DLTS) technique. In addition, the effect of the level of Be-doping concentration on the hole deep traps is investigated. It was observed that with increasing the Be-doping concentration from 1 × 10^16 ^to 1 × 10^17 ^cm^-3 ^the number of detected electrically active defects decreases for samples grown on (311)A substrate, whereas, it increases for (100) orientated samples. The DLTS measurements also reveal that the activation energies of traps detected in (311)A are lower than those in (100). From these findings it is expected that mobilities of 2DHGs in Be-doped GaAs-GaAlAs devices grown on (311)A should be higher than those on (100).

## Introduction

High index planes have attracted a great deal of attention for the production of high quality epitaxially grown semiconductor materials. In particular, the incorporation of silicon as an amphoteric dopant in AlGaAs [1,2] and GaAs [3] grown on high index GaAs substrates have been studied extensively using Hall, photoluminescence and photothermal ionisation measurements. Compared to silicon, beryllium (Be) can be incorporated only as *p*-type dopant in molecular beam epitaxy (MBE) GaAs [4,5] and liquid phase epitaxy grown AlGaAs [6]. Photoluminescence studies have been carried out by Galbiati et al. [7] to investigate the effect of Be incorporation and higher hole mobility in MBE grown *p*-type AlGaAs on (100) and (311)A GaAs orientations. Their results favour (311)A orientation to have more incorporation efficiency and carrier mobility than that of (100) plane. This is due to higher substitutional Be incorporation efficiency in (311)A. It was concluded that good quality *p*-AlGaAs material can be grown on (311)A substrate using Be dopant. Furthermore, it was also reported that the PL spectra of the samples grown on (100) are affected due to the presence of non-radiative centres compared to those grown on (311)A plane. In the light of the above experimental studies, it is important to study and characterise the electrically active deep level defects present in Be-doped AlGaAs grown on (100) and (311)A.

In this study the electrical properties of the defects have been investigated using deep level transient spectroscopy (DLTS) [8], and high-resolution Laplace deep level transient spectroscopy (LDLTS) [9]. These are very powerful techniques to study nonradiative centres. Our electrical experimental studies demonstrate that the numbers of electrically active hole traps in highly Be-doped (311)A AlGaAs layers are less than those observed in (100) devices. The photoluminescence and Hall measurements by Galbiati et al. [7,10] in similar AlGaAs samples show that (311)A samples have higher hole mobilities and well resolved PL spectra than (100) samples. This enhancement of charge mobility and better PL efficiency was suggested to be due to a reduction of electrically active hole traps in (311)A epilayers as compared to those grown on (100) substrates. Our finding is a direct confirmation of their argument.

### Experimental details

A set of six AlGaAs samples with different Be-doping concentrations grown by MBE on semi-insulating (100) and (311)A GaAs substrates have been studied. The samples, labelled as NU1362-NU1367, are described in Table 1. Detailed growth conditions and layer specifications are given in references [7,10].

**Table 1:** Trap parameters calculated from DLTS and Laplace DLTS spectra

Sample ID	Substrate Type	Intensional Doping **(cm**^**-3**^**)**	Trap	**Activation Energy(eV)**	Capture Cross-Section **(cm**^**2**^**)**	Trap Concentration **(cm**^**-3**^**)**	Poole-Frenkel Constant ** (α**_**PF**_**) × 10**^**-5 **^**[(eV)**^**2 **^**cm/V]**^**1/2**^
NU1362	(100)	1 × 10^16^	H_A1_	0.041 ± 0.002	8.32 × 10^-15^	2.09 × 10^13^	10.5
			H_A2_	0.145 ± 0.006	5.35 × 10^-13^	2.74 × 10^13^	27.3
			H_A3_	0.406 ± 0.006	1.89 × 10^-13^	1.67 × 10^14^	-
NU1363	(311)A	1 × 10^16^	H_B1_	0.014 ± 0.006	1.03 × 10^-15^	9.83 × 10^14^	2.2
			H_B2_	0.017 ± 0.004	1.56 × 10^-16^	7.85 × 10^14^	-
			H_B3_	0.305 ± 0.006	5.84 × 10 ^-16^	1.74 × 10^13^	4.2
			H_B4_	0.400 ± 0.003	3.92 × 10^-10^	7.35 × 10^13^	-
			H_B5_	0.430 ± 0.003	1.49 × 10^-12^	3.24 × 10^14^	-
NU1364	(100)	3 × 10^16^	H_C1_	0.356 ± 0.013	1.45 × 10^-14^	1.37 × 10^13^	7.7
			H_C2_	0.383 ± 0.003	8.32 × 10^-13^	8.01 × 10^13^	6.2
			H_C3_	0.403 ± 0.004	8.32 × 10^-13^	8.01 × 10^13^	-
			H_C4_	0.554 ± 0.007	2.29 × 10^-13^	7.68 × 10^13^	-
NU1365	(311)A	3 × 10^16^	H_D1_	0.013 ± 0.001	1.58 × 10^-16^	1.43 × 10^14^	2.0
			H_D2_	0.450 ± 0.004	2.49 × 10^-13^	3.42 × 10^14^	-
NU1366	(100)	1 × 10^17^	H_E1_	0.021 ± 0.002	3.84 × 10^-19^	2.88 × 10^13^	-
			H_E2_	0.130 ± 0.005	1.38 × 10^-18^	4.69 × 10^13^	-
NU1367	(311)A	1 × 10^17^	H_F1_	0.028 ± 0.004	3.83 × 10^-15^	8.47 × 10^13^	-

Schottky contacts were made by evaporating Ti/Au on the top of AlGaAs layer. Top layer has been etched up to 600 nm for the deposition of ohmic contacts [Au/Ni/Au] which were annealed at 360°C in H_2_/Ar mixture.

The deep level defects present in the samples were characterised electrically using DLTS and LDLTS techniques.

## Results and discussion

**Figure 1:**
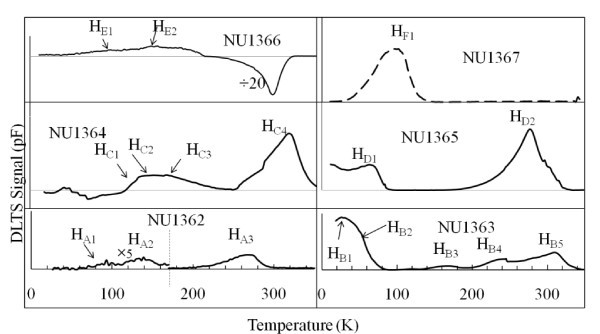
**Conventional DLTS scans for each MBE grown AlGaAs sample**.

**Figure 2:**
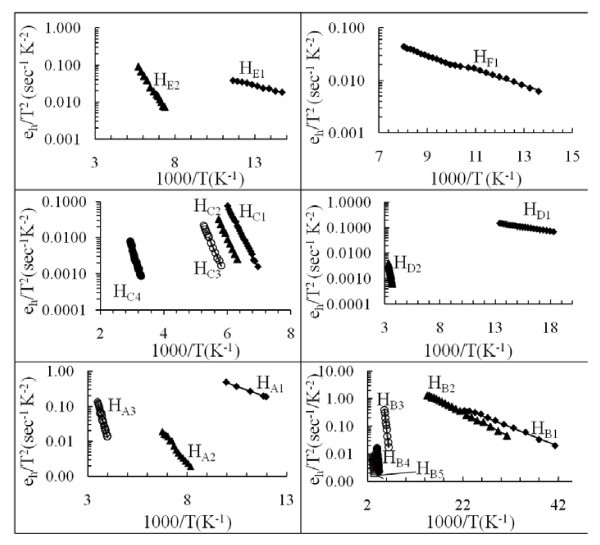
**Arrhenius plot for each hole trap is obtained from Laplace DLTS measurements**. Subscripts A, B, C, D, E and F refer to samples NU1362, NU1363, NU1364, NU1365, NU1366 and NU1367, respectively.

DLTS spectra shown in Figure 1 are obtained using a rate window of 50 Hz, quiescent reverse bias *V*_r _= -3 V, filling pulse *V*_p _= -0.5 V and filling pulse duration *t*_p _= 1 ms. Three and four hole traps are observed in the samples grown on (100) plane for doping concentrations of 1 × 10^16 ^and 3 × 10^16 ^cm^-3^, respectively. In addition to two hole traps, two electron traps are observed in the sample doped to 1 × 10^17 ^cm^-3^. Whereas for the (311)A orientation, five, two and one hole traps have been detected in samples doped with 1 × 10^16^, 3 × 10^16 ^and 1 × 10^17 ^cm^-3^, respectively. In contrast with the (100) samples no electron emitting levels were found in (311)A samples. For convenience holes traps are labelled as H_A_, H_B_, H_C_, H_D_, H_E _and H_F_, in NU1362, NU1363, NU1364, NU1365, NU1366 and NU1367, respectively. The digits correspond to a particular trap in each sample as referred to in Figure 2 and Table 1. Similarly, the detected electron traps are named as E_1 _and E_2_.

High resolution LDLTS [9] technique is used to resolve the broad DLTS peaks obtained by conventional DLTS method. Using the carrier emission rate obtained from LDLTS data by employing equation [8]; $e_{\text{h}}=\left(\frac{\sigma_{\text{h}}\langle V_{\text{th}}\rangle N_{\text{D}}}{g}\right)\exp\left(-\Delta E/kT\right)$ in which <*V*_th_> is carrier average thermal velocity, *N*_D _effective carrier density, *k *is Boltzmann constant and *g *is the trap degeneracy (charge state of the traps after carrier emission), the activation energy of each observed trap (Table 1) is calculated from the slope of an Arrhenius plot of ln(*e*_h_/*T*^2^) versus (1000/*T*) (Figure 2). Here *e*_h _is hole emission rate.

**Figure 3:**
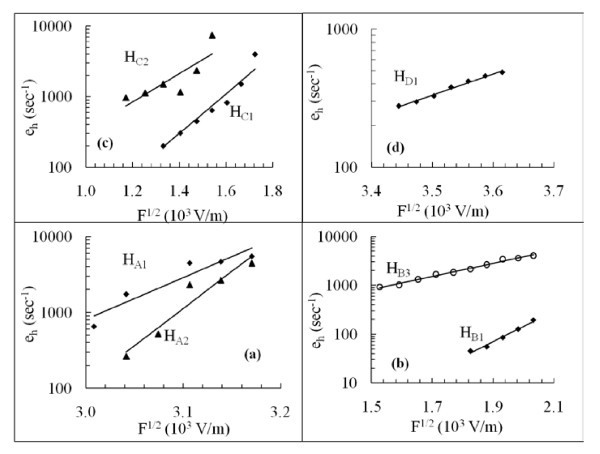
**Traps showing electric field-dependent emission rates**. The data are analysed using Poole-Frenkel model.

For analysis purposes, the trap energies are compared with published data. It is found that the traps H_A2 _and H_E2 _(0.145 ± 0.006 and 0.130 ± 0.01 eV), respectively, have almost the same activation energy as that of H_1 _(0.14 eV) [11], but seem to be different in nature than that of H_1_. For example the capture cross-section of H_1 _[11] was found to be temperature-dependent, whereas in this study the capture cross-sections of H_A2 _and H_E2 _are temperature insensitive. However, H_A2 _shows electric field-dependent emission rate and obeys the Poole-Frenkel model (Figure 3) with constant α_PF _= 10.5 × 10^-5 ^eV(cm/V)^1/2 ^whereas, the carrier emission rate of H_E2 _are electric field-independent.

Similarly, traps H_A3_, and H_B4 _(0.406 ± 0.006 and 0.400 ± 0.003 eV) have similar activation energy as that of H_3 _(0.4 eV) [11]. A broad DLTS peak appeared within the temperature range 130-190 K and is resolved into three different peaks H_C1 _(0.356 ± 0.013 eV), H_C2 _(0.383 ± 0.003 eV) and H_C3 _(0.403 ± 0.003 eV) using Laplace DLTS technique.

The energy of trap H_B3 _(0.305 ± 0.006 eV) is comparable to the activation energy of trap H_3 _(0.30 eV) [12], but H_B3 _found in this study shows an enhancement of the emission rate with the junction electric field. Therefore, it is difficult to confirm that this trap has the same nature.

Traps H_B5 _and H_D2 _(0.430 ± 0.003 and 0.450 ± 0.004 eV) show about the same ground state activation energy as that of H_4 _(0.46 eV) [11]. Another trap H_C4 _(0.554 ± 0.005 eV) has exactly the same activation energy as H_5 _(0.55 eV) [12] with higher capture cross-section and concentration. It is identified as Cu-related trap in MBE grown *p*-type AlGaAs [12].

In addition to the above deep traps some new shallow levels within lower temperature range are obtained in this study, namely H_A1_, H_B1_, H_D1_, H_E1 _and H_F1 _with activation energies 0.041 ± 0.002, 0.014 ± 0.006, 0.013 ± 0.001, 0.021 ± 0.002 and 0.028 ± 0.004 eV, respectively. H_A1_, H_B1 _and H_D1 _show a change in their emission rate with applied bias, whereas, the emission rate for traps H_E1 _and H_F1 _does not change with electric field.

To investigate the effect of the junction electric field on the hole traps emission rate, the LDLTS double pulse method [13] is employed. The difference between two pulse heights is kept constant during each measurement. Considerable change in emission rate of the traps H_A1_, H_A2_, H_B1_, H_B3_, H_C1_, H_C2_, H_D1 _with respect to different filling pulse height is observed. The field-dependent emission rate data are analysed using Poole-Frenkel model [14] as shown in Figure 3. Our experimental data for the traps that obey the Poole-Frenkel model, and the calculated value of Poole-Frenkel constant for each trap are given in Table 1.

This study reveals that the number of traps, including some electron emitting deep levels, increases with increasing Be-doping for the samples grown on (100) plane. On the other hand, the number of hole traps decreases with increasing Be-doping concentrations for (311)A samples. These results are in agreement with the optical studies [7,10] where it was shown that superior PL efficiencies are obtained in Be-doped AlGaAs samples grown on (311)A substrates. The appearance of negative peaks in the samples grown on (100) plane for higher doping level is probably due to residual unintentionally background Si-doping [15]. All the samples used in this study were grown under the same experimental conditions except the variation of Be-doping concentration. The existence of electron traps in the samples grown on (311)A plane is not expected because silicon behaves as a *p*-type dopant on A-faces [1,2].

Investigation of the effect of the electric field on carrier emission rate is one of the useful measurements that give information about the nature of the defect. Electric field-dependent emission rate measurements are carried out and the data are analysed using Poole-Frenkel and phonon-assisted tunnelling models following the simple criteria given by Ganichev et al. [16] to differentiate between both mechanisms. It is evident that the obtained emission rate satisfies the Poole-Frenkel model (Figure 3) with the calculated Poole-Frenkel coefficients (Table 1). This suggests that the emission rate is enhanced due the lowering of Coulomb potential surrounding the defect centre. This also suggests that the defect centres carry no charge when they are filled, and become charged when empty. The nature of the traps before and after the emission can be summarised as *C*^0 ^→ *C*^- ^+ *C*^+^, where *C*^0 ^is the charge state of the defect when it is filled, *C*^- ^is defect charge state when it emits a hole, and *C*^+ ^is the carrier (hole in this case) that is emitted by the trap. Following this argument we are confident to confirm that hole traps found in this study H_A1_, H_A2_, H_B1_, H_B3_, H_C1_, H_C12 _and H_D1 _are acceptor like traps [11,12].

## Conclusion

In summary, we studied the effect of different Be-doping concentrations in AlGaAs layers grown on (100) and (311)A GaAs substrates. It is found that for (100) samples the number of hole traps increases for doping level from 1 × 10^16 ^to 3 × 10^16 ^cm^-3^. In addition, electron emitting levels are detected in samples doped to 1 × 10^16 ^cm^-3^. Detailed studies are required to find out the trap parameters and nature of these negative defects. These electron traps are considered to be due to some Si residual dopant in the MBE system. For (311)A samples the number of hole traps decreases with increasing doping level. It is obvious from the electric field-dependent studies that both charged and neutral like traps exist in the samples. The traps showing the effect of electric field on the carrier emission rates are ionised after carrier emission and carry an electric charge. Finally few shallow level traps are reported for the first time in Be-doped AlGaAs grown by MBE, some of which have an electric field-dependent emission rate. Further studies are needed to explore the nature and origin of these defects.

## Abbreviations

2DHGs: two-dimensional hole gases; DLTS: deep level transient spectroscopy; LDLTS: Laplace deep level transient spectroscopy; MBE: molecular beam epitaxy.

## Competing interests

The authors declare that they have no competing interests.

## Authors' contributions

RHM carried out DLTS and LDLTS measurements, prepared figures and wrote the first draft. MS, MA, AK and MH participated in the analysis of the data and the preparation of the manuscript. MH grew the MBE samples and DT processed the devices.
